# Vesicle-Transported Multidrug Resistance as a Possible Therapeutic Target of Natural Compounds

**DOI:** 10.3390/ph17101358

**Published:** 2024-10-11

**Authors:** Salvatrice Rigogliuso, Alessandra Cusimano, Lucia Condorelli, Manuela Labbozzetta, Gabriella Schiera, Paola Poma, Monica Notarbartolo

**Affiliations:** Department of Biological, Chemical, and Pharmaceutical Sciences and Technologies (STEBICEF), University of Palermo, Viale delle Scienze, 90128 Palermo, Italy; salvatrice.rigogliuso@unipa.it (S.R.); alessandra.cusimano01@unipa.it (A.C.); lucia.condorelli@unipa.it (L.C.); manuela.labbozzetta@unipa.it (M.L.); gabriella.schiera@unipa.it (G.S.); monica.notarbartolo@unipa.it (M.N.)

**Keywords:** cancer drug resistance, P-glycoprotein, extracellular vesicles, acute myeloid leukemia, breast cancer

## Abstract

Background/Objectives: A key role of extracellular vesicles (EVs) is mediating both cell–cell and cell–stroma communication in pathological/physiological conditions. EVs from resistant tumor cells can transport different molecules like P-glycoprotein (P-gp), acting as a shuttle between donor and recipient cells, resulting in a phenotypic change. The aim of our work was to isolate, characterize, and inhibit the release of EVs in two multidrug resistance (MDR) cancer models: MCF-7R (breast cancer cell line) and HL-60R (acute myeloid leukemia cell line). Methods: The existence of P-gp in EVs from MDR cells was confirmed by Western blotting assays. The characterization of EVs was carried out by evaluating the size using NTA and the presence of specific markers such as CD63, Hsp70 and Syntenin. The ability of HL-60R and MCF-7R to perform horizontal transfer of P-gp via EVs to sensitive cells was assessed using three different methods. The acquisition of resistance and its inhibition in recipient cells was confirmed by MTS 3-(4,5-dimethylthiazol-2-yl)-5-(3-carboxymethoxyphenyl)-2-(4-sulfophenyl)-2H-tetrazolium (MTS) assay. Results: Our data showed that cell lines (MDR) release P-gp-loaded EVs, unlike sensitive cells. The acquisition of resistance determined by the incorporation of P-gp into the membrane of sensitive cells was confirmed by the reduced cytotoxic activity of doxorubicin. Natural compounds such as curcumin, lupeol, and heptacosane can block vesicular transfer and restore the sensitivity of HL-60 and MCF-7 cells. Conclusions: Our study demonstrates that natural inhibitors able to reverse this mechanism may represent a new therapeutic strategy to limit the propagation of the resistant phenotype.

## 1. Introduction

Cancer treatment still represents a demanding challenge also due to the development of resistance to chemotherapy. Multidrug resistance (MDR) can be defined as the ability of cancer cells to obtain resistance to both conventional and novel chemotherapy agents. In particular, acquired resistance, which occurs after exposure to chemotherapy, represents an important obstacle to the survival of patients who develop this phenotype, resulting in poor prognosis [1,2].

It occurs that when a cancer cell acquires the resistant phenotype, it also becomes resistant to antitumor drugs characterized by structures and mechanisms of action different from the drug that generated the resistance [3]. Relapse or resistance to chemotherapy is often caused by a selection of clones or some resistant subpopulations of tumor cells that could contribute to recurrence after stopping treatment.

The mechanisms of resistance are multifactorial, and the high expression of ABCB1 (ATP Binding Cassette Subfamily B Member), which encodes the P-glycoprotein (P-gp) [4,5,6] in tumors is associated with high cell survival due to evasion of apoptosis, considerable metastatic potential and extensive extrusion of chemotherapeutic drugs [7].

It has recently been proposed that tumor cell-derived extracellular vesicles (EVs) may be involved in drug resistance [2,8]. Specifically, the transfer of EVs produced by cells with a resistant phenotype to cells with a drug-sensitive phenotype is a mechanism that increases drug resistance and has also been described from preclinical and clinical studies in different types of cancer: lung, breast, gastric, prostate, pancreatic, ovarian, colorectal, melanoma, glioblastoma, osteosarcoma, neuroblastoma, kidney, and hematological malignancies [8,9,10,11,12,13].

EVs are particles also released by tumor cells into the extracellular space. Based on their biogenesis and release mechanisms, EVs are classified into exosomes, microvesicles, and apoptotic bodies. Exosomes originate from endosomal compartments with a diameter between 40 and 150 nm. Microvesicles originate by budding outward from the plasma membrane, with a diameter between 200 and 1000 nm. Apoptotic bodies are released during programmed cell death, with a diameter greater than 1 µm [14,15].

The role of vesicular cargo is particularly decisive, and in this context, the existence of P-gp has been demonstrated, which contributes to the influx of drugs into EVs by maintaining their intracellular concentration at a sublethal level [16,17].

It has been described that only MDR cells produce exosomes expressing P-gp compared to their sensitive counterparts, consistent with the fact that it is present only in MDR cell models in several tumor types [18,19,20,21].

There is much evidence regarding the crucial role of cancer-derived EVs in the transmission of biological signals between different populations of cells. In different types of tumors, EVs are involved in several stages of the oncogenic process, from tumor initiation to tumor progression, metastasis and chemotherapy failure [22]. The EVs obtained from the plasma of cancer patients presented proteins/glycoproteins on cell membranes and/or in the cytosol of the parent tumor cells [14]. EVs derived from resistant breast and leukemia cancer cells transfer P-gp and confer MDR to recipient cells [18,23].

For all these reasons, EVs play a key role in regulating both the close microenvironment and far sites, contributing to the tumor’s ability to grow, invade and metastasize. The inhibition of EV secretion and uptake plays a significant role among the different mechanisms contributing to chemoresistance. The pharmacological approaches implemented both in vitro and in vivo target blocking vesiculation and remove the produced EVs. Several categories of molecules, as well as proton pump inhibitors, calpain inhibitors, MEK inhibitors and others such as vitamins or antibacterial and antifungal, interfere with the vesicle formation process in the tumor cell but, unfortunately, also on the physiological one. On the contrary, removing the “pathological” vesicles using specific markers present only on tumor-derived EVs would avoid interference with normal physiological processes [24,25,26,27,28,29].

In this work, we intend to further underline the importance of blocking the spread of P-gp-mediated drug resistance and explore the possible chemosensitizing effects of natural compounds that act as inhibitors of the vesiculation process, in two different multidrug-resistant cell models of breast cancer (MCF-7R) and acute myeloid leukemia (HL-60R). To our knowledge, the only natural compound capable of inhibiting the process of vesicle release from doxorubicin-resistant breast cancer cells is psolaren, a furanocoumarin [30].

Previous studies conducted on MDR models demonstrated how curcumin, lupeol, and heptacosane are able to restore the sensitive phenotype by acting as P-gp blockers. In fact, they stimulate the ATPase activity of P-gp by behaving as inhibitory substrates, and only for curcumin and lupeol, also determine a reduction of the protein expression [31,32,33]. Our previous knowledge about these molecules and their action in acquired resistance mechanisms, especially P-gp-mediated mechanisms, led us to explore whether these substances could also target the acquisition of resistance mediated by extracellular vesicles.

## 2. Results

### 2.1. Multidrug-Resistant Cells Produce More EVs than Their Sensitive Cellular Counterparts

**Figure 1:**
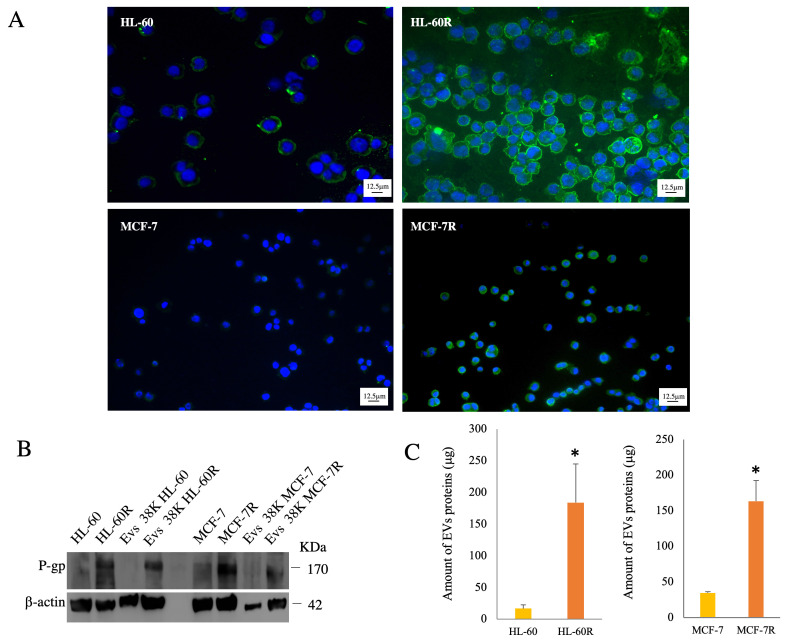
Evaluation of P-gp expression in EVs isolated from resistant cell lines. (**A**) Immunofluorescence analysis of HL-60, HL-60R, MCF-7, and MCF-7R cells. Nuclei (DAPI Blue); P-gp (green). (**B**) Western blotting analysis of cell extract and 38,000 rpm fractions (38K) of EVs isolated, respectively, from HL-60R and MCF-7R cell lines. (**C**) Comparison of the amount of protein content in EVs released from both sensitive cell lines compared to resistant cell lines. Data were normalized to the same number of cells: HL-60R vs. HL-60 * *p* < 0.05; MCF-7R vs. MCF-7 * *p* < 0.01 (Tukey’s test).

**Figure 2:**
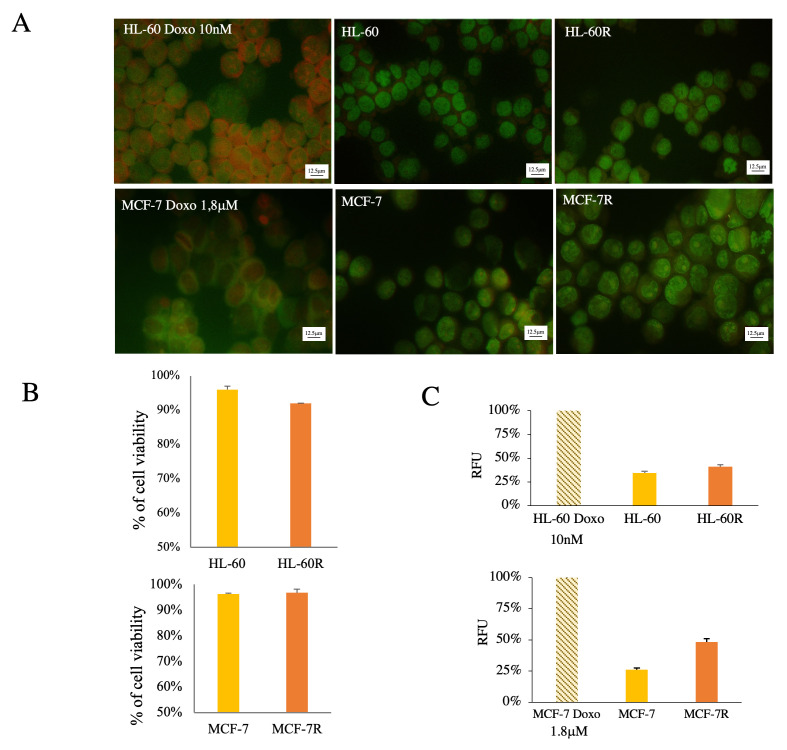
Analysis of cells producing EVs (38K) with AO staining: (**A**) fluorescence microscopy images; (**B**) Trypan Blue exclusion assay; (**C**) spectrofluorometric analysis. Fluorescence emission of the cells was compared to positive control (apoptosis induced by doxorubicin treatment; RFU—Relative Fluorescence Units).

We have isolated by ultracentrifugation and characterized EVs from the MDR models previously developed of acute myeloid leukemia HL-60R and breast cancer MCF-7R (According to MISEV 2023). Previous studies have highlighted how both resistant cell lines are characterized by the overexpression of unfavorable biological and pharmacological factors, such as the constitutive activation of NF-κB and the overexpression of inhibitory apoptosis proteins (IAPs) and P-gp on the cell surface (Figure 1A), involved in the MDR phenotype [34,35]. Western blotting analysis detected high P-gp expression only in EVs released by MDR cell lines (Figure 1B) because this protein is only present in the MDR models. Furthermore, it was possible to observe that EVs isolated from the same amount of live cells contain a greater number of total proteins in MDR cells compared to sensitive cells (Figure 1C). The presence of apoptotic bodies was excluded with a viability assay using acridine orange (AO) dye for fluorescence microscopy (Figure 2).

**Figure 3:**
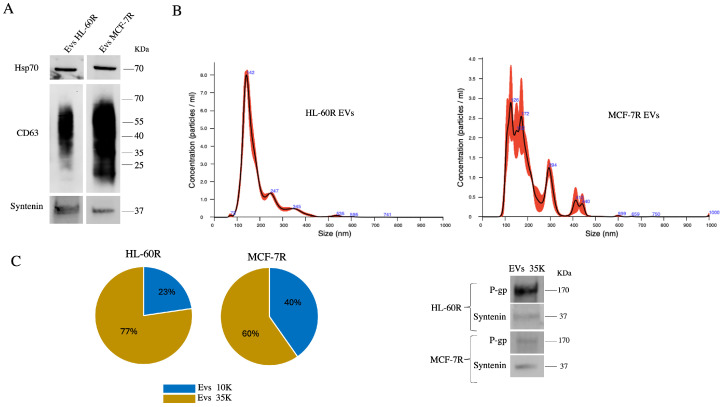
Characterization analysis of EVs isolated from resistant cell lines. (**A**) Western blot analysis of the EV markers, Hsp-70, CD63, and Syntenin. (**B**) Size distributions of EV fractions isolated, respectively, from HL-60R and MCF-7R cell lines measured by nanoparticle tracking analysis (NTA). (**C**) Separation of heterogeneous EV populations by differential ultracentrifugation and relative %; Western blotting analysis for P-gp and Syntenin expression in a 35k EV fraction.

We have characterized EVs from MDR cells in terms of the presence and size of EV markers such as CD63, Hsp70 and Syntenin (Figure 3A). The isolated populations of EVs were analysed for their size by nanoparticle tracking analysis (NTA), and the results showed heterogeneous populations of EVs characterized by sizes ranging from 70 to 200 nm (Figure 3B). By differential ultracentrifugation, we obtained different populations demonstrating that vesicles of different sizes are present, probably microvesicles and exosomes, the latter present in greater percentages (77% in HL-60R cells and 60% in MCF-7R cells, respectively) and which express P-gp and Syntenin (Figure 3C).

### 2.2. MDR Cells Are Able to Carry Out Horizontal Transfer of P-gp through the Release of EVs onto Sensitive Cells

**Figure 4:**
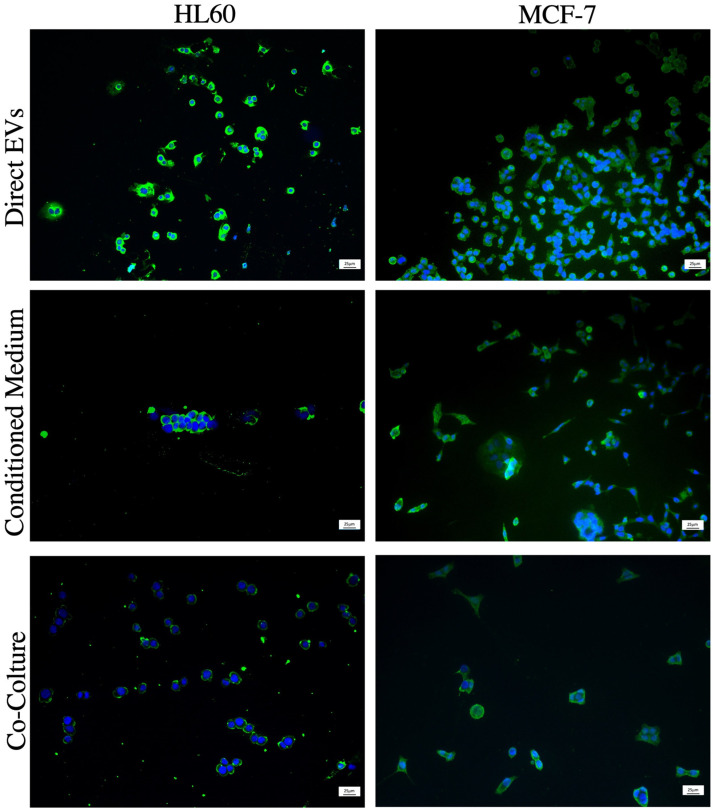
Analysis of the horizontal transfer of resistance. Representative image of evaluation of P-gp expression after following treatment of the sensitive HL-60 and MCF-7 cell lines: EVs isolated from the respective resistant cell lines, with the medium conditioned by the cells of the respective resistant lines or by the co-culture growth of the sensitive and resistant line in a Transwell plate. Nuclei (DAPI Blue); P-gp (green); magnification 20× and scale bar was 25 µm.

The ability of HL-60R and MCF-7R to carry out the horizontal transfer of P-gp through the release of EVs on sensitive cells was verified by evaluating three different transfer methods: treatment with conditioned medium deriving from the culture of the MDR cells, treatment with pure extract of vesicles produced by resistant cells and co-culture of sensitive-resistant cells. Immunofluorescence analysis with anti-P-gp antibody demonstrated how the transfer occurs for both tumor models and with all the methods used (Figure 4). These experiments were performed at different times: we observed that P-gp transfer from pure EVs occurs after 8 h of exposure, from the conditioned medium after 24 h and from resistant cells in co-culture after 48 h and 24 h for the HL-60 and MCF-7 cells, respectively.

### 2.3. Natural Compounds Are Able to Reverte Resistance Acquisition via Vesicles

**Figure 5:**
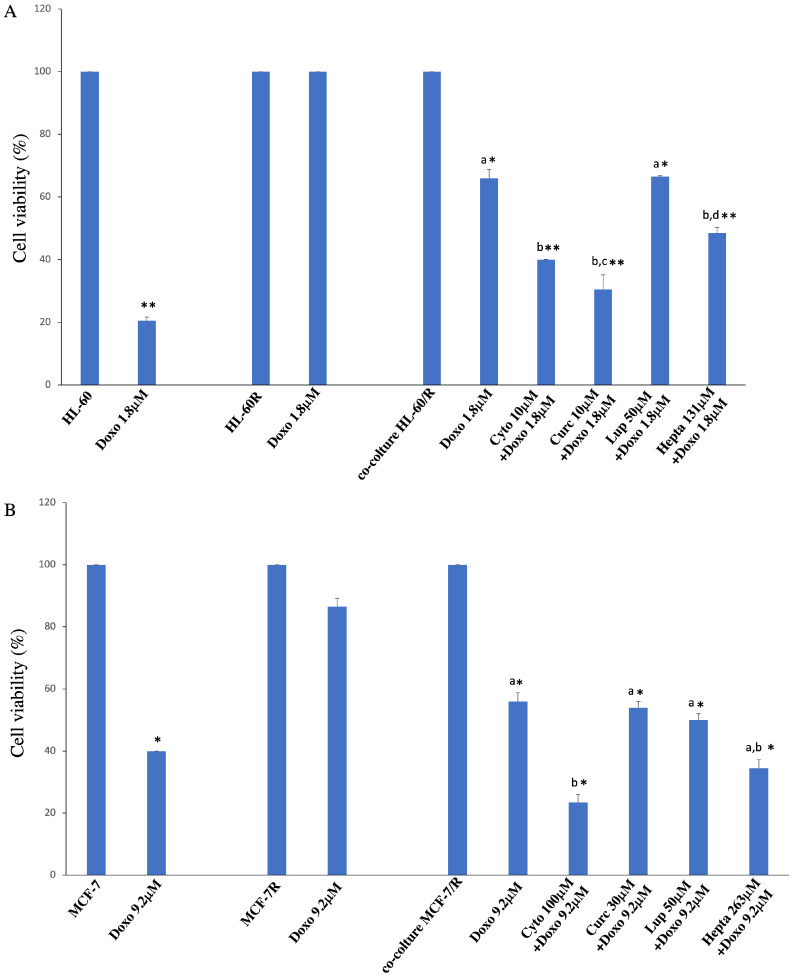
(**A**) Cells were exposed to Doxorubicin (1.8 μM) for 16 h after co-culture. Cell viability was assessed by an MTS assay. Letters indicate significant differences (Tukey’s test) in cell viability among the concentrations of each cell line (Cytochalasin, Curcumin, and Heptacosane vs. co-culture + Doxorubicin *p* < 0.005; Cytochalasin vs. Lupeol *p* < 0.001; Curcumin vs. Lupeol and Heptacosane *p* < 0.05). Treatments are likened to the control: * *p* < 0.005; ** *p* < 0.001. (**B**) Cells were exposed to Doxorubicin (9.2 μM) for 24 h after co-culture. Cell viability was assessed by cell counting. Letters indicate significant differences (Tukey test) in cell viability among the concentrations (Cytochalasin and Heptacosane vs. co-culture + Doxorubicin *p* < 0.05; Cytochalasin vs. Lupeol and Curcumin *p* < 0.05). Treatments are likened to the control: * *p* < 0.05.

To investigate whether vesicle-transported P-gp is functional and causes a phenotypic change in sensitive cells and to study the reversal of resistance acquisition determined by natural substances, we conducted a co-culture experiment. The resistant cells seeded into the cell culture insert were treated with a known vesiculation inhibitor, cytochalasin D, and with natural substances lupeol, curcumin, and heptacosane at the concentrations indicated. Table 1 reports the relative IC_50_ values. Subcytotoxic concentrations were extrapolated from the viability curves obtained on both cell models. Only curcumin in the MCF-7R cell line induced a reversal of the acquisition of resistance visible at the concentration corresponding to its IC_50_. We performed a cytotoxicity assay after doxorubicin exposure. As shown in Figure 5A,B, in both cases, we deal with a reversal of acquired resistance significantly determined by curcumin and heptacosane in the leukemia cell model and by heptacosane in the breast cancer cell line.

### 2.4. P-gp Transfer Occurs in 3D Breast Cancer Model

**Figure 6:**
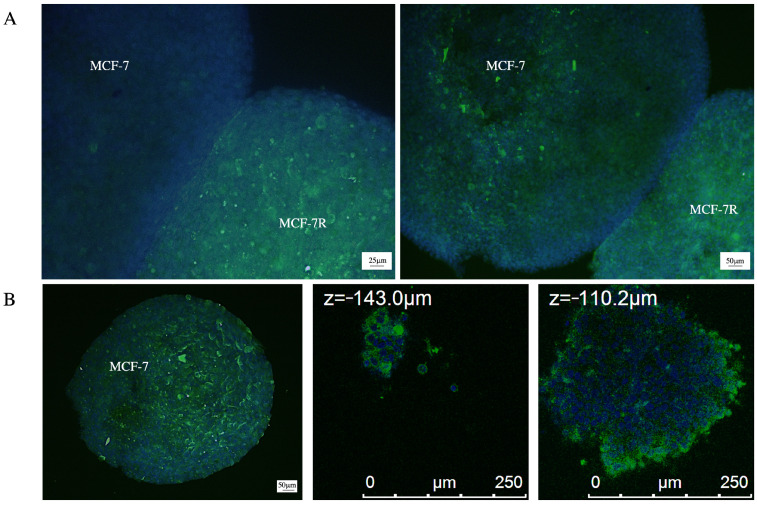
Co-culture of MCF-7 and MCF-7R cell spheroids. MCF-7 and MCF-7R cell spheroids in co-culture for 24 h, labeled with DAPI (blue nuclei) and anti-P-gp mAb (green fluorescence). (**A**) Immunofluorescence at time zero and after 24 h of co-culture with an anti-P-gp antibody; magnification 60× and scale bar was 25 µm. (**B**) Confocal microscopy with Z-stack imaging; magnification 40× and scale bar was 50 μm.

To evaluate whether P-gp transfer is a mechanism that can be observed in a model that mimics key features of human solid tumors, we produced spheroids from sensitive and resistant MCF-7 cells and grown in co-culture. After 24 h, it is possible to observe a high expression of P-gp in the spheroid of sensitive cells with respect to the control at time zero, indicating that the P-gp transfer could also occur via the EVs in 3D models (Figure 6A). Z-stack analysis under confocal microscopy reveals that P-gp expression is also found in more internal regions of the spheroid and not only on the outer surface (Figure 6B).

## 3. Discussion

MDR is frequently observed in patients with leukemia and breast cancer treated with different drug regimens currently applied in the clinical practice, and one of the causes has been associated with the acquisition of high P-gp expression [1,2,3,4,5,6,7]. P-gp is an ATP-dependent drug transporter localized on the surface of tumor cells that is up-regulated in drug-resistant tumors. EVs have a dual role in the phenomenon of drug resistance. Scientific evidence from studies of preclinical and clinical samples associates EVs with the transmission of anti-cancer drug resistance from cell to cell in multiple cancer types [9].

In particular, EVs transfer P-gp and other transporters by fusion with the cell membranes of susceptible clones in several human cancer models, such as prostate and ovarian cancers, acute T lymphoblastic leukemia, and osteosarcoma [2]. Gong et al. demonstrated that resistant breast cancer cells release microparticles in which the P-gp is oriented oppositely to that present in the cells from which they originate [36]. Sequestration of chemotherapeutic agents in intracellular vesicles and in compartments far from the cellular target, such as lysosomal drug compartmentalization, may also contribute to MDR [37]. EVs can mediate drug resistance by directly exporting or sequestering cytotoxic drugs, reducing their concentration at target sites. In a breast cancer cell line, it was shown that the chemotherapy drug doxorubicin was physically encapsulated in vesicles and expelled into the extracellular medium [24]. To counteract drug resistance, it is important to understand how sensitive cell clones undergo a phenotypic change.

We confirmed the presence of P-gp in two drug-resistant models selected in our laboratory by exposure to increasing doses of doxorubicin and demonstrated that MDR tumor cells release more EVs than their drug-sensitive counterparts, and these vesicles contain P-gp like the MDR cells that generated them. Scientific evidence from studies of preclinical and clinical samples associates EVs with the transmission of anti-cancer drug resistance from cell to cell in multiple cancer types [9].

We characterized the vesicles released from the two MDR cell models by size and observed the expression of markers such as CD63, Hsp70 and Syntenin proteins present in exosomes and related to their biogenesis and/or release [38,39]. It is known that exosomes are found in all body fluids, and their cargo signature can be used to predict cancer type as well as stage and outcome in terms of therapeutic result [40,41].

The transport of P-gp via the vesicles to sensitive cells and the acquisition of the resistant phenotype can certainly be considered one of the mechanisms of acquired resistance. This acquired resistance can be counteracted, for example, by using inhibitors that prevent the propagation of the resistant phenotype. We have previously demonstrated that natural compounds such as curcumin, lupeol, and heptacosane are able to inhibit P-gp through different mechanisms at the transcriptional and post-transcriptional levels, blocking its efflux function and modulating its ATPase activity [42]. For this reason, we compared the effects of these natural compounds to those of Cytochalasin D. In fact, it was reported that cytochalasin D, due to its mechanism of action, could be considered an inhibitor of both exosome release and endocytic processes. Cytochalasin D is a natural alkaloid that inhibits the elongation phase of actin polymerization by binding to the edges of actin filaments, preventing subunit association or dissociation, thus inhibiting actin polymerization. Given the importance of actin in the cytoskeleton reorganization for trafficking towards the cell membrane, cytochalasin D may possibly be used to prevent EV release [43,44,45].

In this study, we demonstrated that curcumin and heptacosane are able to reverse P-gp transfer-mediated doxorubicin drug resistance and cause a phenotypic change in sensitive AML cells and heptacosane in breast cancer cell models. We hypothesized a mechanism of action similar to that of cytochalasin D, which is able to destroy the formation of an organized network. This hypothesis requires further investigation. Preliminary experiments were useful to observe that vesicle-mediated transport of P-gp from resistant to sensitive cells could also occur in a 3D co-culture breast cancer model.

Interestingly, tumor cells in vivo are able to release EVs not only into the bloodstream but also into tissue fluids. The development of experimental models able to mimic this process requires further advance and investigation.

EVs have a potential diagnostic role, as they could be considered not only as valuable biomarkers for early diagnosis and surveillance of various malignancies but also as markers of drug response prediction and tumor disease recurrence [10,46]. For example, it would be interesting to evaluate whether, in patients with innate resistance, there are circulating vesicles carrying factors implicated in drug resistance or whether they increase during therapy.

## 4. Materials and Methods

### 4.1. Cell Lines and Culture

The human breast adenocarcinoma cell line MCF-7 and the human acute myeloid leukemia cell line HL-60 were obtained from ATCC, Manassas, VA, USA, whereas their variants, MCF-7R and HL-60R, respectively, were selected for MDR by exposure of the susceptible cell lines to gradually increasing concentrations of doxorubicin in our laboratory. HL-60 and HL-60R cells were cultured in Roswell Park Memorial Institute (RPMI) 1640, while MCF-7 were cultured in Dulbecco’s Modified Eagle Medium (DMEM). Both media were supplemented with 10% heat-inactivated fetal calf serum, 2 mM l-glutamine, 100 U/mL penicillin, and 100 μg/mL streptomycin. Cells were cultured in a humidified atmosphere at 37 °C in 5% CO_2_.

### 4.2. Extracellular Vesicle Isolation

To isolate extracellular vesicles from the conditioned medium, the MCF-7R and HL-60R cells were grown in 175 cm^3^ flasks in 25 mL of complete medium/flask (four flasks for each preparation) until reaching 80% confluence, then were placed in serum-free medium for 24 h. The cell-conditioned medium was then recovered in 50 mL Falcon tubes and centrifuged first at 1500 rpm for 10 min, then at 3000 rpm × 15 min, to eliminate cell debris. After this centrifugation step, the supernatant was collected in Ultraclear Beckman tubes and ultracentrifuged at 38,000 rpm × 2 h at 4 °C (called 38K) in a Ti60 rotor (Optima L-90K Ultracentrifuge, BECKMAN, Brea, CA, USA). Vesicles recovered by ultracentrifugation were resuspended in 40 µL of PBS and quantified by Qubit fluorometer and Qubit™ Protein Assay Kit (Invitrogen™ A50669, Thermo Fisher Scientific, Waltham, MA, USA). To collect the different populations of vesicles separately, two cycles of ultracentrifugation were carried out on the serum-free conditioned medium, recovered from the respective cell cultures after 24 h of culture: 10,000 rpm × 30 min (called 10K) 35,000 rpm × 1 h 30 min (called 35K). The samples were quantified using a Qubit fluorometer, and the number of different populations of EVs isolated was evaluated. The viability of the cells from which the vesicles were isolated was assessed by the Trypan Blue exclusion test.

### 4.3. Immunofluorescence Analysis

Cytospin preparations were used to concentrate tumor cells in a given area. A concentration of 2 × 10^4^ cells/200 µL at 1000 rpm for 5 min was used to transfer cells onto poly-lysine-coated slides. Cells were fixed with methanol cold at −20 °C (15 min), permeabilized with 0.1% Triton-X 100/PBS (10 min) and blocked with 3% BSA in PBS for 15 min at room temperature. After blocking, the cells were incubated with anti-P-glycoprotein (clone F4, mouse monoclonal IgG1, Thermo Fisher, working dilution 1:100) in humid chamber overnight at 4 °C after washes slides were incubated for 1 h at room temperature with the secondary fluorescence-labeled antibody goat anti-mouse Fitc (Invitrogen, working dilution 1:400) and counterstained with DAPI and mounted with a coverslip. Images were acquired with a fluorescent microscope (DM2500, Leica, Wetzlar, Germany) equipped with a camera (DFC450 C, Leica, Wetzlar, Germany).

### 4.4. Western Blotting Analysis

The HL60 and HL-60R cell lines grown in suspension were washed in PBS. However, MCF-7 and its resistant variant MCF-7R were washed in PBS and detached from the culture flask mechanically with a scraper, then centrifuged at 1000 rpm for 5 min at 4 °C. The pellet of each sample was resuspended in RIPA Lysis Buffer (sc-24948 Santa Cruz Biotechnology, Dallas, TX, USA) (*v*/*v*) and incubated for 60 min in an ice bath. After centrifugation (14,000 rpm, 10 min, 4 °C), the protein content in supernatants was determined using Bradford reagent (Quick Start Cat.500-0205 BIO-RAD, Hercules, CA, USA) in a plate reader at 595 nm and calculated based on a BSA standard curve.

The cell extracts and their respective vesicles directly placed in the buffer under reducing conditions were subjected to 4–15% polyacrylamide gel electrophoresis in SDS-PAGE at 100 Volts for 1 h. The gel-resolved protein pathway of each sample was transferred to a membrane (Hybond-P nitrocellulose, GE Healthcare Europe GmbH, Freiburg, Germany). The filters were incubated with 5% BSA to saturate nonspecific sites present on the samples, and after with primary antibodies in 1% BSA for 24 h at 4 °C with stirring: P-gp (mAb Ms 1:200 MA5-13854 Invitrogen, Thermo Fisher Scientific, Waltham, MA, USA), CD63 (mAb Rb 1:1000 E1W3T Cell Signaling, Danvers, MA, USA), Hsp70 (mAb Ms 1:1000 sc-7298 Santa Cruz Biotechnology, Dallas, TX, USA) MDA9/Syntenin (SDCBP mAb Ms 1:500H00006386-M01 Abnova, Taipei City, Taiwan) as housekeeping were used β-actin (mAb Ms 1:5000 SAB1305554 Sigma-Aldrich, St. Louis, MO, USA) and GADPH (mAb Ms 1:10.000 G8795 Sigma-Aldrich, Srl, Milan, Italy). Membranes were further incubated with the relevant secondary antibodies linked to Horseradish Peroxidase for the detection of the proteins under examination: (ECL-anti Mouse IgG-HPR NA931V 1:2500 Amersham, Slough, Buckinghamshire, UK) and (ECL-anti Rabbit IgG-HPR NA934V 1:2500 Amersham, UK) in 1% BSA for 2 h at room temperature with stirring. Hybridization was visualized by Versa DOC (BioRad Laboratories, Hercules, CA, USA) using a chemiluminescence detection kit (SuperSignal West Femto Maximum Sensitivity Substrate, Thermo Scientific Life Technologies). Immunoblots were quantified by densitometry, and the results were expressed as arbitrary units (protein/β-actin or protein/GADPH).

### 4.5. Acridine Orange Staining

Cells from which the conditioned medium for EV isolation was recovered were centrifuged at 1000 rpm for 10 min, counted, and then 400.000 cells of each line (HL-60/R and MCF-7/R) were resuspended in 200 µL of a 100 µg/mL Acridine Orange (AO) solution. The relative fluorescence emission was immediately read by spectrofluorimeter (Ex.485/20nm-Em.590/35) and compared to the fluorescence emission of positive control, in which apoptosis was induced by treatment for 24 h with a sub-cytotoxic concentration of doxorubicin (10 nM for HL60 and 1.8 µM for MCF-7).

For the microscopy observation, cells were attached to the cover glass using a cytocentrifuge. Shortly thereafter, 400,000 cells of each line were resuspended in 500 µL of 4% paraformaldehyde and maintained for 1 h at room temperature. After the fixation step, the cells were washed three times in 1 mL of PBS by centrifugation at 1000 rpm for 5 min/wash. 200 µL of the cell solution were deposited on a polylysinated slide through two different cycles of centrifugation in a cytocentrifuge: 1000 rpm for 5 min and subsequently 1800 rpm for 1 min. The slides thus prepared were immersed in a 100 µg/mL AO solution for 10 s, washed in abundant PBS, and immediately observed under a fluorescence microscope.

### 4.6. Nanoparticle Tracking Analysis (NTA)

The NTA Nanoparticle Tracking Analysis technique uses the properties of light scattering and Brownian motion to obtain the size distribution and concentration of samples in liquid suspension. The size and concentration of EVs were measured by NTA using a Nanosight LM10 (Malvern Instruments, Worcestershire, UK) equipped with a 488 nm laser. The camera level was automatically adjusted, and the analysis detection threshold was set at 3–4. EV samples were diluted in filtered PBS to obtain optimal concentrations (20–80 particles per frame). Three video recordings were carried out for each EV preparation. Nanosight NTA 3.3 software (Malvern Instruments) was used to analyze the recorded video. The results obtained by the NTA technology are the size distribution, the relative scattering intensity of each particle, the fluorescence of each particle, and the total concentration of the sample.

### 4.7. Cell Culture with EVs

MCF-7 cells were seeded at 5 × 10^3^ /well into eight-well chamber slides Nunc Lab-Tek II CC2 Chamber Slide System, and EVs derived from MDR cells at a concentration of 20 µg/mL were added for different time points (4, 8, and 24 h). Also, in these cases, the cell monolayers were fixed and then stained for immunofluorescence.

HL-60 cells were seeded at 1 × 10^5^ in 12-well plates, and after reaching the growth confluence, EVs derived from MDR cells at a concentration of 20 µg/mL were added for different time points (4, 8 and 24 h). After these times, a cell suspension was recovered in the Eppendorf safe lock tube of 1.8 mL and centrifuged at 1200 rpm for 7 min. The conditioned medium was discarded and the cell pellet was resuspended in fresh RPMI. The cells were centrifuged with Cytospin on polysine slides for immunofluorescence staining.

### 4.8. Cell Culture in Conditioned Medium

MCF-7 cells were seeded at 5 × 10^3^/well into eight-well chamber slides (Nunc Lab-Tek II CC2 Chamber Slide System). As soon as they attached themselves, the DMEM medium was removed and replaced with a conditioned medium recovered from the MCF-7R cells grown at confluence for different time points (8, 24, and 48 h). After these time periods, the MCF-7 cells were fixed and processed for immunofluorescence staining.

HL-60 cells were seeded at 1 × 10^5^ into a 12-well plate, and after reaching the growth confluence, RPMI medium was removed and replaced with conditioned medium recovered from HL-60R cells grown at confluence for different time points (8, 24, and 48 h). After these times, a cell suspension was recovered in an Eppendorf safe lock tube of 1.8 mL and centrifuged at 1200 rpm for 7 min. Then, the conditioned medium was discarded and the cell pellet was resuspended in fresh RPMI. The cells were centrifuged with Cytospin on polysine slides for immunofluorescence staining.

### 4.9. Cell Co-Culture

MCF-7 cells at 1 × 10^5^ were seeded into a six-well plate. The cell culture inserts were placed into the six wells, and the MCF-7R cells were seeded into the inserts (1 × 10^5^ cells/insert). After 24, 48, and 72 h, the inserts were removed, and the MCF-7 cells were trypsinized. The MCF-7 cells at 2 × 10^3^ in 200 μL were centrifuged with Cytospin at 1000 rpm for 5 min on polysine slides (Epredia Italia s.r.l., Milan, Italy) and fixed in cold methanol for immunofluorescence staining.

HL-60 cells at 1 × 10^5^ were seeded into a six-well plate. The cell culture inserts were placed into the six wells, and the HL-60R cells were seeded into the inserts (1 × 10^5^ cells/insert). After 24, 48, and 72 h, the inserts were removed, and the HL-60 cells were recovered in the Eppendorf safe lock tube and centrifuged at 1200 rpm for 7 min. Then, the supernatant was discarded, and the cell pellet was resuspended in fresh RPMI. The HL60 cells at 2 × 10^3^ in 200 μL were centrifuged with Cytospin at 1000 rpm for 5 min on polysine slides and fixed in cold methanol for immunofluorescence staining.

### 4.10. Evaluation of the Reversal of Acquired Resistance

The sensitive cells were plated in a 1:1 ratio with the resistant cells plated into cell culture inserts of 0.4 µm (Sigma Aldrich srl, Milan, Italy) and treated with different compounds (the MCF-7R cell line for 24 h and HL-60R cell lines for 48 h). After these times, the inserts were eliminated, and sensitive cells with acquired resistance via vesicles from resistant cells, both untreated and treated with vesiculation inhibitors, were exposed to doxorubicin (1.8 µM for 16 h for the HL-60 and 9.2 μM for 24 h for MCF-7). Contextually, parental and MDR cell lines were treated with doxorubicin at the same times and concentrations. We performed the cytotoxicity assay with a 3-(4,5-dimethylthiazol-2-yl)-5-(3-carboxymethoxyphenyl)-2-(4-sulfophenyl)-2H-tetrazolium (MTS, Promega Corporation Madison, WI, USA) assay for the HL-60 cells and a Trypan Blue exclusion assay for MCF-7 cells.

### 4.11. Cancer Spheroid Culture and Immunofluorescence

The MCF-7 and MCF-7R cells were grown in DMEM complete medium in ultralow attachment plates (Corning Inc., New York, NY, USA). On day 0, 3000 cells with collagen I (6 μg/mL) were seeded into each well of the plates.

Plates were centrifuged for 3 min at 1000 rpm. After 24 h, 100 μL of complete medium was added into each well, and the plates re-centrifuged. After 4 days, the MCF-7 spheroids were co-cultured with MCF-7R spheroids and, after 24 h, were fixed in 4% paraformaldehyde, incubated with anti-P-gp and stained with DAPI. After blocking, the cells were incubated with anti-P-glycoprotein (clone F4, mouse monoclonal IgG1, Thermo Fisher, working dilution 1:100) in a humid chamber overnight at 4 °C. Afterward, the washed slides were incubated for 1 h at room temperature with the secondary fluorescence-labeled antibody, Goat Anti-Mouse FITC (Invitrogen, working dilution 1:400), counterstained with DAPI, and mounted with a coverslip. Images were acquired with a fluorescent microscope (Leica DM2500) equipped with a camera (Leica DFC450 C) and using a Leica TCS SP5 confocal laser scanning microscope and a 63 × 1.4 oil objective (Leica, Wetzlar, Germany).

## 5. Conclusions

Research on the mechanisms that govern exosome cargo selection and formation is in its early stages, and specific inhibitors of these processes are not yet known. These inhibitors may regulate exosome secretion and have exhibited promising results in the investigation of new therapies to target drug resistance phenotypes. Moreover, EVs could be used as drug delivery systems thanks to their ability to encapsulate and release their cargo into the extracellular space [15].

## Figures and Tables

**Table 1:** IC_50_ values of indicated compounds in the two MDR cell lines.

	HL-60R (IC_50_ ± SE)	MCF-7R (IC_50_ ± SE)
Cytochalasin D	17.0 ± 5.3 µM	>500.0 µM
Curcumin	13.0 ± 0.7 µM	30.0 ± 0.3 µM
Lupeol	>100.0 µM	>100.0 µM
Heptacosane	>263.0 µM	>263.0 µM

## Data Availability

All data are available upon request from the corresponding author.
